# Molecular and epigenetic regulatory mechanisms of normal stem cell radiosensitivity

**DOI:** 10.1038/s41420-018-0132-8

**Published:** 2018-12-18

**Authors:** Maria Rita Fabbrizi, Kacie E. Warshowsky, Cheri L. Zobel, Dennis E. Hallahan, Girdhar G. Sharma

**Affiliations:** 1 Cancer Biology Division, Department of Radiation Oncology, Washington University School of Medicine, 4511 Forest Park, Saint Louis, MO 63108 USA; 2 Siteman Cancer Center, Washington University School of Medicine, Saint Louis, MO 63108 USA

## Abstract

Ionizing radiation (IR) therapy is a major cancer treatment modality and an indispensable auxiliary treatment for primary and metastatic cancers, but invariably results in debilitating organ dysfunctions. IR-induced depletion of neural stem/progenitor cells in the subgranular zone of the dentate gyrus in the hippocampus where neurogenesis occurs is considered largely responsible for deficiencies such as learning, memory, and spatial information processing in patients subjected to cranial irradiation. Similarly, IR therapy-induced intestinal injuries such as diarrhea and malabsorption are common side effects in patients with gastrointestinal tumors and are believed to be caused by intestinal stem cell drop out. Hematopoietic stem cell transplantation is currently used to reinstate blood production in leukemia patients and pre-clinical treatments show promising results in other organs such as the skin and kidney, but ethical issues and logistic problems make this route difficult to follow. An alternative way to restore the injured tissue is to preserve the stem cell pool located in that specific tissue/organ niche, but stem cell response to ionizing radiation is inadequately understood at the molecular mechanistic level. Although embryonic and fetal hypersensity to IR has been very well known for many decades, research on embryonic stem cell models in culture concerning molecular mechanisms have been largely inconclusive and often in contradiction of the in vivo observations. This review will summarize the latest discoveries on stem cell radiosensitivity, highlighting the possible molecular and epigenetic mechanism(s) involved in DNA damage response and programmed cell death after ionizing radiation therapy specific to normal stem cells. Finally, we will analyze the possible contribution of stem cell-specific chromatin’s epigenetic constitution in promoting normal stem cell radiosensitivity.

## Facts

- Ionizing radiation is a common cancer treatment, but it is often accompanied by side effects which cause normal tissue injuries and a decline in the quality of life.
- Radioprotective drugs have been proven effective in vitro but fail to replicate their effect in vivo; the only FDA-approved drug available, Amifostine, is currently used to reduce xerostomia but it has also been proven to offer protection against several chemotherapeutic agents.
- The loss of the stem cell pool is believed to be the cause of the normal tissue injuries and stem cells have been proven to be highly radiosensitive compared to differentiated cells.
- Stem cell radiosensitivity is regulated by pluralistic mechanisms that involve both epigenetic and molecular signaling. Improved understanding of the regulatory pathways that make stem cells radiosensitive would lead to innovative radioprotective drug development and novel therapies to eradicate cancer while preserving the stem/progenitor cells.

## Open questions

- Do stem and non-stem cells respond differently to DNA breaks?
- Are stem cells epigenetically programmed to favor cell death instead of repair and survival after radiation exposure?
- What are the molecular mechanisms involved in the stem cell radiosensitivity?

## Introduction

Following induction of DNA damage, cells respond in different ways and this DNA damage response (DDR) depends on several variables, such as cell cycle, post-translational modifications of the signaling cascade, and chromatin configuational changes^1–3^. When the DNA strand break is not severe or irreparable, cells respond by activating DNA repair pathways. Double-strand break repair is achieved by two major DNA repair pathways: homologous recombinational repair pathway (HR) which operates only in the post-replicative S or G2/M phases of cell division cycle and requires a homologous sister chromatid and non-homologous end joining (NHEJ) which operates mostly in the pre-replicative G1 phase of the cell cycle and is the most prominent form of DNA repair mechanism in terminally differentiated cells. When the damage is irreparable, cells respond with cell cycle arrest, apoptosis, senescence, or several other cell mechanisms^4,5^.

Ionizing radiation (IR) therapy is commonly used to treat cancers with the aim of inducing DNA double-strand breaks (DSBs) in cancer cells. The use of radiation therapy to kill cancer cells also causes DNA damage in the surrounding normal tissue and patients who undergo IR exposure experience treatment-related symptoms during therapy, months or even years after. Early side effects include erythema, dry desquamation, intestinal malabsorption, hyperpigmentation, and hair loss^6–8^. Late effects include skin atrophy, dryness, telangiectasia, dyschromia, dyspigmentation, fibrosis, ulcers, and neurocognitive decline^9–12^. Many decades ago it was perceived that a single stem cell was able to partially replenish the physiology of IR-damaged tissues^13,14^ and lack of this cell pool can lead to different side effects, such as accelerated aging, cognitive impairment, and poor learning and memory, especially in pediatric brain cancer patients. Stem cells at the pluripotent stage are capable of self-renewal and can produce all undifferentiated cell types of the tissue of origin, serving as an internal repair system by dividing and replenishing/replacing the dead cell populations. Because of their ability to restore damaged tissue, research has been driven towards the pathway of stem cell transplantation: although the only approved stem cell transplant in clinical practice is the bone marrow transplant used for cancers affecting the blood or immune system such as leukemia, lymphoma, or multiple myeloma, many other fields have been explored and recent findings suggest stem cell transplantation may provide a useful intervention strategy for minimizing the adverse effects of several pathologies such as Parkinson’s disease, amyotrophic later sclerosis, diabetes mellitus, heart disease, and much more^15–20^. However, application of stem cell transplant therapy to alleviate cognitive deficits in CNS malignancy treatment regimen has enormous practical limitations. Therefore, in loco stem cells could restore the normal physiology for tissues that have been injured by IR, given that the radiation did not cause any DNA damage in those cells^21,22^. Current studies in the literature are, however, inconclusive regarding the capability of DNA repair in stem cells after IR exposure, with several studies suggesting stem cells are more prone to activate apoptotic response pathway instead of DDR^23–30^.

This review will give an overview of normal tissue injury after radiation exposure and normal stem cell radiosensitivity to elucidate the developments in known mechanisms underlying radiation response in order to find a suitable pharmacological approach to protect stem cells from radiation-induced cell death.

## Tissue injury after irradiation and known mitigation/radioprotection agents

The response of normal tissues to radiotherapy differs from one another after a chemical, thermal, or mechanical stress: radiation therapy damages DNA, proteins, and lipids in the cell through direct ionization or by production of free radicals, such as reactive oxygen species^31^. It also increases apoptosis, chromosomal aberrations, and mutation frequency in cells^32^. Fluorescence in situ hybridization probes show significant increases in chromosomal aberrations in murine splenocytes after total body irradiation, with a high number of translocations^33^, while in human lymphocytes a radiation dose-dependent increase of micronuclei formation has been observed, with multi-chromosome material detected above 2 Gy^34^.

These injuries can initiate several signaling pathways, including the inflammatory response, eventually leading to cell death, senescence, or disruption of tissue physiological functions^35,36^. Radiation injury-directed therapies can be divided into three groups: prevention of radiation damage by intervention before treatment, injection of a radioprotective agent during or immediately after radiation therapy in order to minimize the development of clinical injury, and treatment of radiation injury after therapy to prevent the progression to clinical impact^37^. Some commonly available treatments have shown promise in prevention or mitigation of normal tissue injury after irradiation, making drug-based approaches important for normal tissue radioprotection. Sulfhydryl (SH) compounds are known for their ability to act as radioprotectors; both cysteine and cysteamine have been proven to protect animals from the effects of total body radiation if they are injected or ingested before the radiation exposure. Sh-mediated cytoprotection has mainly two components: these radioprotectors are free-radical scavenging which are able to withdraw oxygen-based free-radical generation; moreover, their ability to donate hydrogen atoms has the potential of improving DNA repair. The only radioprotective drug approved by the U.S. Food and Drug Administration for use in radiation therapy is amifostine, a non-reactive phosphorothioate which is converted to its active form by the enzyme alkaline phosphatase. This drug has been proven to reduce side effects such as dry mouth and difficulties in eating or speaking in patients with head and neck cancer treated with radiotherapy, but it also showed significant protection against several chemotherapeutic agents. Amifostine, in pratice, is used only for the reduction of xerostomia^38^.

Another treatment of radiation injury after therapy to prevent the progression to clinical impact is stem cell transplantation. It is known that the stem cell pool has the ability to restore normal tissue structure/function: in fact, in 1967 an experiment by Withers elegantly showed that even one stem cell was sufficient to regrow nodules on a confined annulus of irradiated mouse skin^39^. Later on, the technique was perfected to obtain the survival characteristics of crypt cells of the mouse jejunum^40^, testicular stem cells^41^, and kidney tubules^42^. It has also been shown that stem cells are able to produce colonies when transplanted from a donor animal to a different site in a recipient mouse^43,44^. Due to their ability to repopulate an injured tissue, adult stem cells have been proposed for use in the treatment of radiation-induced tissue damage. Hematopoietic stem cell transplantation is currently used to reinstate blood production in leukemia patients, but several pre-clinical treatments with stem cells have shown promising results in different organs (i.e. skin, eye, lung, kidney) and might potentially translate into tissue radioprotection strategies^45^. However, there are many issues regarding use of stem cell therapy, including the lack of definitive markers for stem cells in different tissues, the rarity of the population for a successful transplant, together with major ethical and expense concerns covering the conventional radiotherapy. Therefore, a possible way to avoid complications related to transplant is to find new ways to protect the stem cell pool residing in the irradiated tissue. It is in fact suggested that stem cells are highly sensitive to IR; thus their ability to repopulate an injured tissue could be compromised after radiotheraphy. The mechanisms responsible for stem cell radiosensitivity have not been completely elucidated yet.

## Embryonic and fetal DDR and sensitivity to IR

Radiation exposure of embryos and fetuses is of great concern for radiological diagnostics, radioprotection, and human health. Typical fetal doses from diagnostic radiology are usually low but some radio-diagnostic-specific treatments like cancer radiotherapy can expose the fetus to higher and potentially unsafe radiation doses^46^. The deleterious effect of IR to the embryo or fetus is strongly dependent on the stage of development, the absorbed dose, and fractionation^47–49^. According to extensive studies performed on animal models, a dose of 0.05–0.5Gy at the first and second week of pregnancy will slightly increase the incidence of implantation failure, but surviving embryos will probably have no significant health effects. During early organogenesis (third to sixth week postconception) the embryo is very vulnerable to growth retardation, teratogenic, and lethal effects of high-dose irradiation, while during early fetal stage the fetus develops a higher tolerance to radiation exposure, although the central nervous system can be seriously affected by high levels of IR exposure. At the end of pregnancy, the fetuses generally do not show any malformation after exposure to low amounts of radiation^50^. Moreover, some effects of radiation can only be observed many years post-exposure, such as behavioral disorders, infertility, neoplasia, and shorter life span^51^. Therefore, a definitive trend of hypersensitivity of embryonic cells has been observed in vivo over the past many decades in clinics and in experimental models as well.

However, research on embryonic stem cell in “culture” models have been inconclusive and often contradict the in vivo observations most likely due to late-passage spontaneous transformation effects in culture. It has been reported that DSBs caused by IR exposure in human embryonic stem cells (hESC) lead to the activation of the ataxia telangiectasia mutated (ATM) signaling pathway^52,53^, activating several proteins such as p53, Chk2, and Nbs1. Additionally, the number of γ-H2AX IR-induced foci (IRIF) increases dramatically within minutes following IR and returns almost to control levels in unexposed hESCs cultures within 24 h^54^. Besides the role of ATM in H2AX phosphorylation, Rad3-related (ATR) kinase was also proposed to play a role in the process^53,55^. However, our studies on multiple stem cell tissue niches in vivo and early-passage primary stem and isogenic differentiated non-stem cells have shown that murine embryonic stem cells are unable to activate ATM after IR-induced DNA DSB^24,30^ and that the γ-H2AX IRIF are significantly reduced compared to differentiated counterparts^23^. Similar results were observed by Suchorska and colleagues comparing human embryonic SCs, human induced pluripotent SCs, and primary human dermal fibroblasts^56^. Human embryonic stem cells show a robust apoptotic response after low IR exposure^25,57^ and a dramatic decrease in cell viability^26,56^ in a cell-cycle-dependent fashion^58,59^. Interestingly, the surviving hESCs continued to express common pluripotency markers and embryonic transcription factors, such as Oct4, Sox2, and Nanog^25,52,53,56,60^. The protection of hESCs from apoptosis following different exposures has been investigated and many biological pathways are shown to be involved^61–67^, but there are insufficient details regarding the response of hESCs after IR exposure. DNA synthesis and cell proliferation were partially inhibited^68–71^ but high expressions of anti-apoptotic protein Bcl-2 were found after irradiation together with low levels of pro-apoptotic proteins, indicating that the apoptotic response was not induced after IR exposure^68,72–75^. In contrast, early-passage primary murine embryonic stem cells showed high apoptotic response after 6 Gy exposure compared to their differentiated isogenic progeny^23,24^ together with high levels of pro-apoptotic factor Bax and low levels of anti-apoptotic protein Bcl-2 (ref. ^30^). hESCs show a higher number of aberrant mitotics after 2 Gy dose of irradiation compared with negative control and an arrest of the cell cycle in G(2)/M^52^. The influence of IR exposure on hESCs transcriptional response has been analyzed by Sokolov and colleagues: they found that hESCs have a clear pro-apoptotic transcriptional response 2 h after IR exposure, with upregulation of several genes such as BTG2, CDKN1A, SESN1, IER5, GADD45A which are genes often associated with temporal cell cycle arrest. At 16 h post IR the transcription patterns change, showing a strong expression of genes involved in pro-survival pathways and general metabolic signaling^54^. We observed IR-induced changes in the murine embryonic stem cell transcriptome which were associated with affecting biosynthesis, such as ribosome expression, while the non-stem cell transcriptome promoted activation of survival signaling pathways as well as protein digestion and absorption^30^. This aspect of the IR influence on hESCs has not been scrutinized deeply yet; thus, further studies are required. We therefore believe there is a need of serious evaluation of appropriate stem cell radiosensitivity and DDR research models to establish a common platform for inferring and proposing mitigating and/or preventive measures for radioprotection.

### Adult stem cells sensitivity to IR

Adult stem cells have the tendency to respond differently from embryonic stem cells after IR-induced DNA damage. While hESC preferentially undergo apoptosis after IR treatment, adult stem cells exhibit a broad variety of responses and studies of stem cell radiosensitivity/radioresistance in literature are generally inconclusive (reviewed in refs. ^76,77^). A possible explanation is that stem cells in different tissues reside in niches that have tissue-specific environmental factors needed for stemness maintenance; moreover, the cell cycle status seems likely to play a role. Most importantly, the stem cell models used for studying the phenomenon or the mechanism is crucial in drawing inferences. It is in fact known that some adult stem cells are in the G0 phase of the cell cycle and are therefore quiescent. Many factors intervene in the maintenance of this status, which is believed to be important to preserve long-term proliferative potential and genomic integrity^78,79^. However, as quiescent cells, adult stem cells lack DNA damage checkpoints and are unable to activate cell cycle-dependent repair pathways, thus genomic integrity is not maintained^80–83^.

### DDR activation

Human mesenchymal stem cells (hMSCs) are believed to be relatively resistant to IR exposure^84–87^. The proposed explanation for hMSCs is that their niche in vivo is hypoxic, so hMSCs may already be able to hinder the IR effects by having signaling machinery capable of responding to several insults at normoxic conditions^88^. DDR, like ATM protein phosphorylation, cell-cycle checkpoint activation, and DSB repair are some of the cellular mechanisms proposed to explain hMSCs radioresistance^72,85,89,90^. Some studies observed high levels of phosphorylated histone H2AX in MSC after IR exposure as a marker of DNA DSB repair process^74,85–87,91,92^, while other studies found overexpression 3 days post IR indicating a possible execution of cell senescence program^68^. Recently, it has been proposed that human MSCs exposed to prolonged X-irradiation accumulated γ-H2AX and 53BP1 foci differently compared to acute X-irradiation^93^; a careful distinction between the roles of apoptosis-related pan-nuclear γ-H2AX versus DDR-related γ-H2AX IRIF specific to the DSBs is required^23^.

After 4 Gy of IR treatment, human hematopoietic stem cells (hHSCs) did not show any activation of the cell cycle checkpoints^29^, but high levels of γ-H2AX have been reported in hHSC subpopulations when compared to more mature progenitors at 12 h post 3 Gy of IR, indicative of persistent DNA DSBs in these cells^94^. These results are in contrast to those presented after non-IR-related oxidative stress induction in hHSCs. It has been shown that hHSCs respond to oxidative damage with a strong activation of ATM, p53, 53BP1, CHK2, and FOXO3a and a senescence-like cell cycle arrest^95,96^. Recently, Biechonski and colleagues^97^ found that hematopoietic stem and early progenitor cells exhibit reduced NHEJ activities in comparison to non-lineage committed progenitors, along with more persistent 53BP1 foci. Moreover, the DNA repair process in hHSCs needs the presence of thrombopoietin, which supports important microenvironmental factors in the regulation of hHSCs IR exposure response^98^. Keratinocyte and melanocyte stem cells have reportedly shown high levels of γ-H2AX IRIF after 5 Gy irradiation with reduced colony-forming activity in culture and delayed hair cycle in vivo^99^ while lower dose of IR did not exert any effect on cell survival^100^.

**Fig. 1:**
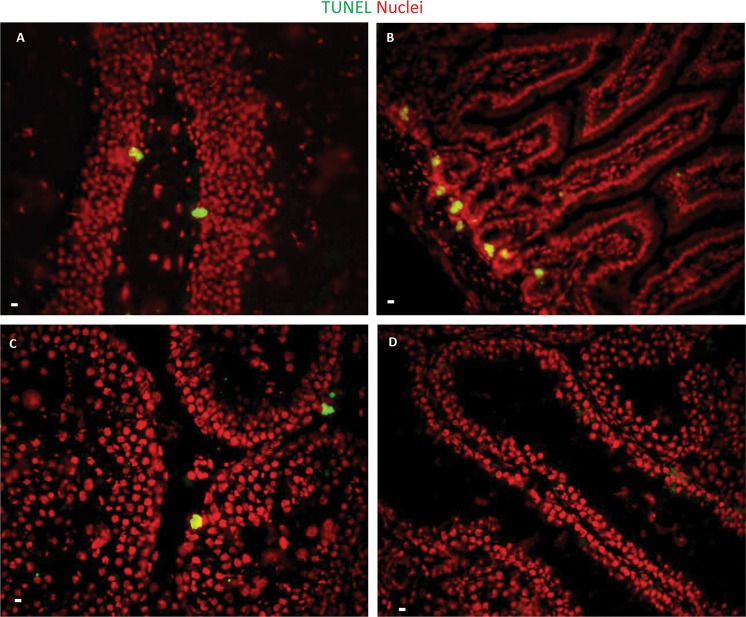
Stem cells are more radiosensitive than their differentiated progeny. **a** TUNEL staining (yellow-green signals) was performed in the dentate gyrus of the hippocampus obtained from brains of C57BL/6 mice after 6 Gy whole-body IR. **b** TUNEL staining was performed in tissue sections of adult murine intestine after 6 Gy IR. **c** TUNEL staining was performed in tissue sections of adult murine testis after 6 Gy IR. **d** TUNEL staining was performed in tissue sections of adult murine testis after 12 Gy IR. Scale bar = 10 µm. Original picture included for illustrative purposes, referring to ref. ^23^

**Fig. 2:**
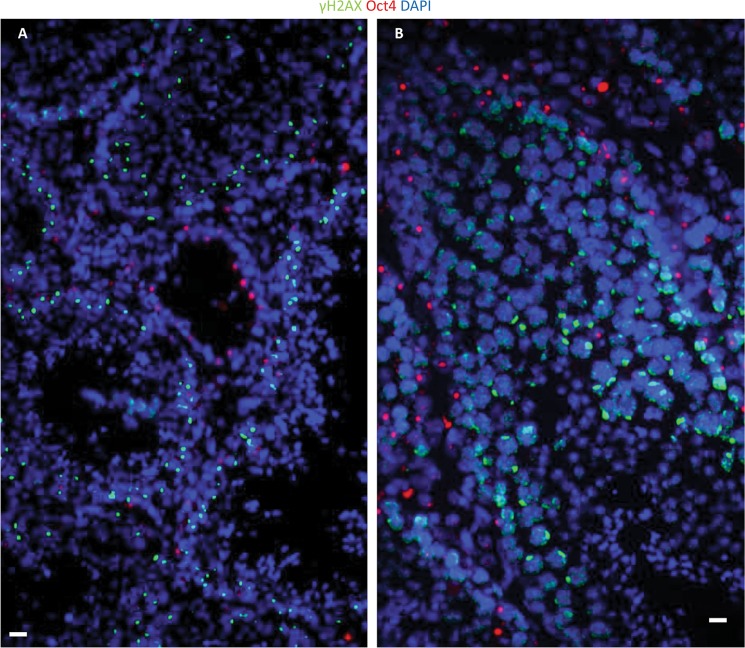
Stem cells (marked with red signals) fail to form γ-H2AX foci (green signals) after ionizing radiation treatment. γ-H2AX was detected in adult murine testis stem cells by immunostaining after 0Gy (A) and 6 Gy IR (B). Scale bar = 10 µm. Original picture included for illustrative purposes, referring to ref. ^23^

Limited studies on DNA DSB repair in human neural stem cell (hNSC) cultures have reported the presence of γ-H2AX foci after irradiation, which reaches the IRIF level of non-IR treated cells 3 h after IR exposure^27^. However, careful in vivo experimentations by our group that has been published in recent years are in contrast with these findings where we demonstrate that instead of foci formation, pan-nuclear H2AX-S139 phosphorylation is observed and that murine neural stem cells selectively undergo IR-induced apoptosis compared to the non-stem differentiated cells in the hippocampal tissue niche (Fig. 1a). Stem cells in intestine (Fig. 1b) and testis (Fig. 1c) showed the same response, with superficial tissues like testis showing massive depletion of spermatogenesis in response to irradiation (Fig. 1d). As mentioned before, careful observation of DSB IRIFs versus pan-nuclear apoptotic γ-H2AX in multiple tissue niches and primary culture models, noγ-H2AX IRIFs were detected in stem cells (Fig. 2), indicating that stem cells are deficient in enabling DDR activation and DNA repair after radiation exposure^23^.

More detailed studies are required to determine whether the varying DDRs observed in several stem cell populations is due to dissimilar cell cycle status, since stem cells from diverse niches proliferate at different rates or may be in a quiescent or proliferative stage.

### Apoptotic response activation

hHSCs respond with massive apoptotic activation following low dose IR, showing both dose and time dependency^28,29,101,102^ and an implication of Bcl-2, p53, and ASPP1 in the process^94^. The same response has been observed in murine HSCs after 2 Gy treatment^30^. The clonogenic potential in 2 Gy-irradiated hHSCs decreased to ∼50–60% compared with the non-irradiated control^103^ while Wang et al.^104,105^ found that 6.5 Gy total body irradiation drove HSC to senescence. On the contrary, Chang and collaborators^106^ found that in vivo 1 Gy exposure was sufficient to drive HSC to quiescence, without any apoptotic effect observed in stem cells. No significant alterations in cellular senescence and apoptosis were detected in HSCs after exposure to low dose of radiation^107^.

**Fig. 3:**
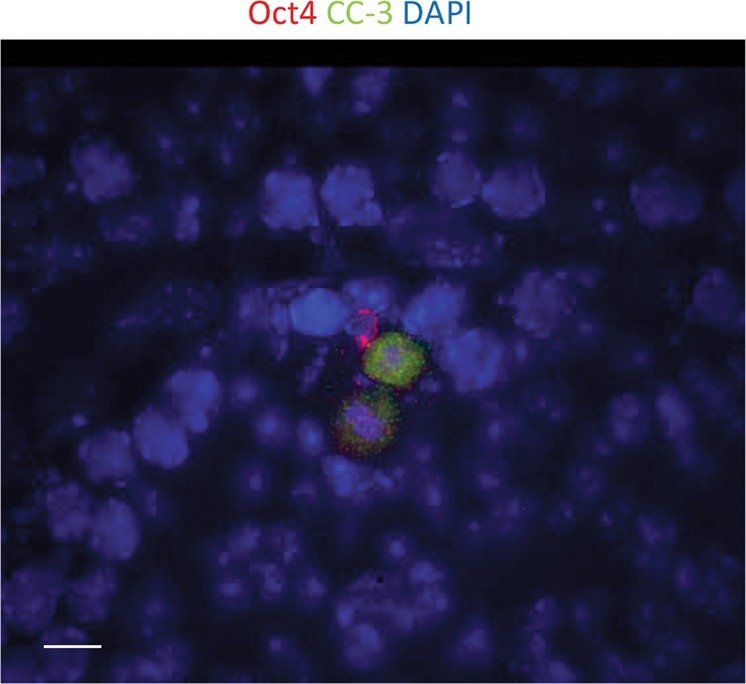
Stem cells undergo apoptosis after ionizing radiation treatment. Cleaved caspase-3 (CC-3) was detected in adult murine testis stem cells by immunostaining after 6 Gy IR. Scale bar = 10 µm. Original picture included for illustrative purposes, referring to ref. ^23^

hNSCs were previously shown to undergo programmed cell death after low, modest, and high doses of IR^24,27,57,108^ in a dose-dependent way^27^, with the surviving populations mostly starting a senescence process^109^, although hNSC radiosensitivity seems to be subpopulation-dependent^110^. Additionally, it has been recently observed that 10 Gy treatment affected both the cell survival and degree of differentiation in NSC through a bystander effect and activation of pro-apoptotic factors^111^. The apoptosis-susceptible nature of the irradiated hNSCs has been associated with a TRAIL-R2-mediated signaling cascade with activation of caspase-3 (ref. ^112^) and with prolonged upregulation of phosphorylated p53 (ref. ^113^). Activation of caspase-3 was found in murine spermatogonial stem cells after 6 Gy IR exposure (Fig. 3) and has been published before^23,24,30^.

P53-dependent apoptosis has also been observed in Lgr5+ intestinal stem cells after irradiation in culture and mice^114^. In vivo experiments on mice show that 10% of intestinal stem cells initiate apoptosis after low doses, although cell death had no appreciable effect on tissue architecture^115,116^, while 15 Gy treatment caused more widespread apoptosis of Lgr5+ intestinal stem cells, resulting in a failure to restore viability of the small intestines^117^. We found Lgr5+ intestinal stem cells in ex vivo organoid cultures undergo massive apoptosis after 6 Gy irradiation while proliferative cells marked with Ki67 positivity largely comprised of SSEA1-negative non-stem cells in the same organoids showed greater radioresistance^30^.

The effect of IR on mesenchymal stem cells has been widely scrutinized in the past: minimal induction of cellular apoptosis was observed after treatment up to 20 Gy, with MSCs showing high expression levels of anti-apoptotic proteins BCL-2 and BCL-XL and low levels of pro-apoptotic proteins such as Puma (reviewed in ref. ^73^).

Although different stem cell populations respond with a diverse degree of programmed cell death, the dose utilized seems to play a fundamental role in the process.

### Cell cycle arrest

hMSCs show a high and constant level of p53 after high doses of gamma-irradiation^68^, with ATM implicated in the post -translational modifications of p53 on ser 15, Chk1 on ser 345, and Chk2 on thr 68 (ref. ^118^). On the contrary, Kurpinski et al.^119^ found a modest gene expression change 5 h post 1 Gy of IR in hMSCs, although the downregulation of cyclin E2 (CCNE2) caused cell cycle arrest at this time point, while Jin et al.^120^ observed a complex response in the transcriptomic analysis of hMSCs after IR exposures, with early-response genes expressed at low doses and late-response gene at the highest doses. These conflicting results can be explained in the elegant work of Wu and colleagues: using early- and late-passage mesenchymal stem cells, they found IR exposure resulted in a decreasing trend in arresting both early- and late-passage MSCs in the G0/G1 phase up to 72 h post IR, and a substantial accumulation of early-passage MSCs in the G2/M phase. These results indicate that early-passage MSCs possess more effective cell cycle checkpoints in G2/M following IR exposure. It is likely that DNA damage is repaired through error-free HR in early-passage MSCs. Given that more late-passage MSCs were in the G0/G1 phase, the error-prone NHEJ can be the major DNA repair mechanism for late-passage MSCs, which may result in more genomic alterations^87^.

Intestinal stem cells undergo massive apoptosis after 20 Gy irradiation and show G2/M arrest induced by radiation to prevent mitotic catastrophe in a p53-independent manner^121^. In contrast, lower doses induced p53-mediated cell cycle arrest and protect the stem cell niche after DNA damage^122^. Lgr5+ intestinal stem cell radiosensitivity has been suggested to be CDK 4/6-dependent^123^, although a more recent study suggested that their radiosensitivity is related to DNA damage-dependent activation of Wnt signaling^124^. Moreover, although small and large intestines possess seemingly similar Lgr5+ stem cells, colonic epithelial stem cells (CESC) have been shown to be markedly more radioresistant in vivo than small intestinal crypt base columnar stem cells. Lgr5+ CESCs displayed delayed checkpoint recovery at 48 h post-19 Gy, which correlated with complete DSB repair and regeneration of colonic mucosa^125^.

It seems therefore that the cell cycle status might play an important role in stem cell radiosensitivity, dictating the activation of error-prone or error-free DNA repair mechanism after IR.

### The pluralistic regulatory mechanisms of stem cell sensitivity to IR

**Fig. 4:**
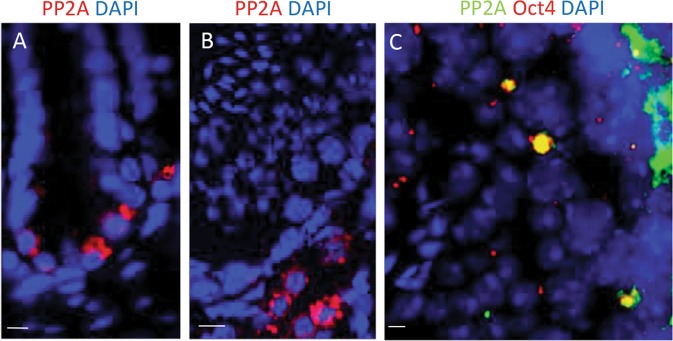
Stem cells display constitutively elevated IR-induced PP2A levels. **a** PP2A detected in adult murine intestine by immunostaining at 0 Gy. **b** PP2A detected in adult murine testis by immunostaining at 0 Gy. **c** PP2A detected in adult murine testis by immunostaining at 30 min after 6 Gy. Scale bar = 10 µm. Original picture included for illustrative purposes, referring to ref. ^30^

Stem cell radiosensitivity is due to several signaling pathways that are activated/deactivated in response to irradiation. We believe that pluralistic interaction of molecular and epigenetic mechanisms collectively regulate and impart IR hypersensitive phenotype to the normal stem cells. Recently, we have found that stem cells constitutively express PP2A, which antagonizes and impairs DDR activation and promotes apoptosis at 0 Gy (Fig 4a, b) and after 6 Gy treatment (Fig. 4c), as has been published before^30^, confirming the hypothesis of Chowdhury et al.^126^.

The method by which gene expression is regulated without altering the genomic sequence, known as epigenetics, is another key factor involved in this multifaceted mechanism. Epigenetic changes include DNA methylation, histone acetylation, and miRNA-regulated gene expression^127,128^. Epigenetic alterations have been found to contribute to the pathogenesis of radiation-induced carcinogenesis^129^ by the reactivation of oncogenes and the silencing of tumor suppressor genes^130^. These events can result in genomic instability and consequent carcinogenesis in many models^131–134^.

Many epigenetic studies on ESC and induced pluripotent SC maintenance and differentiation have been extensively reviewed^135–140^, while little is known about epigenetic modifications after IR exposure.

**Fig. 5:**
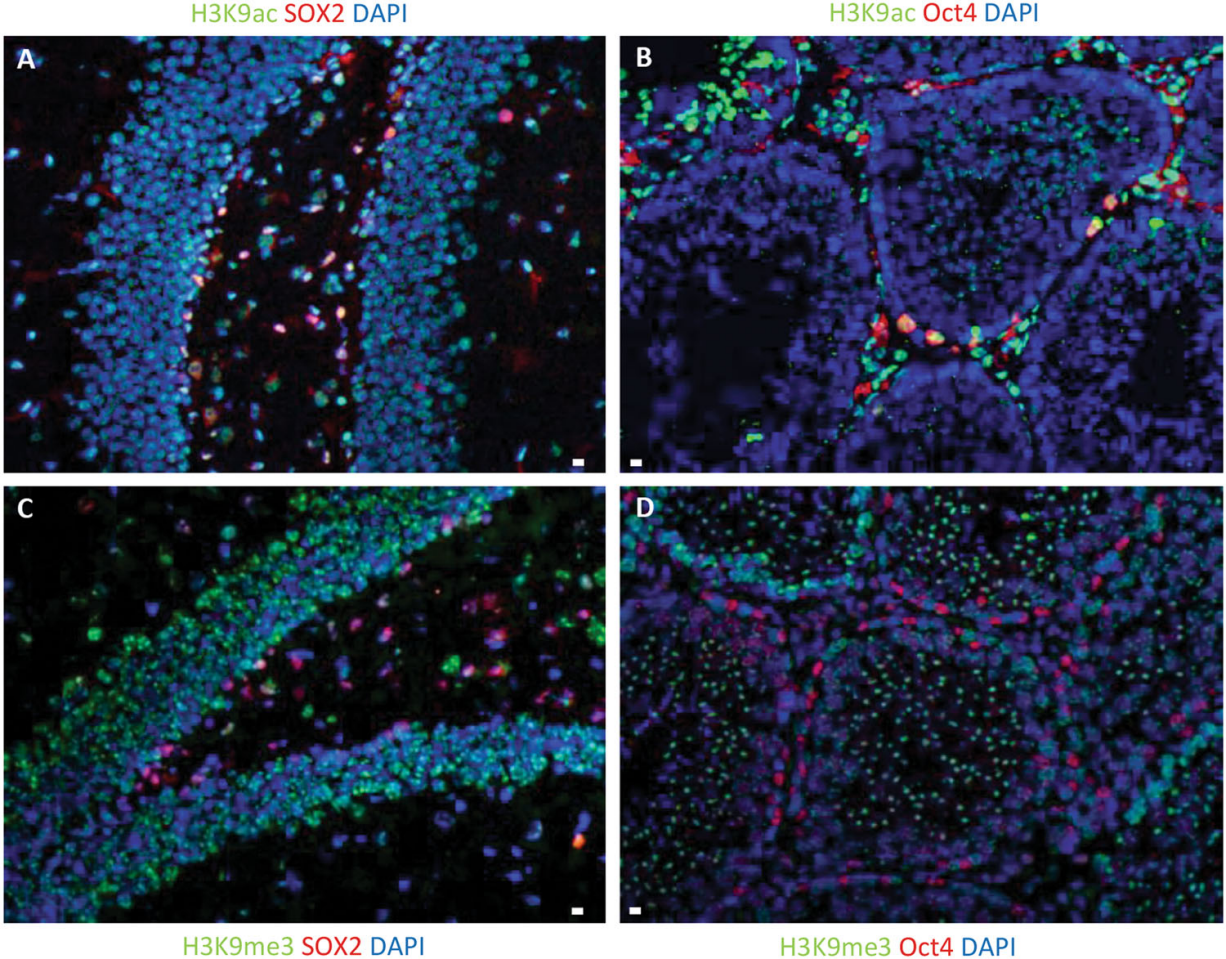
Stem cells fail to deacetylate H3K9 after ionizing radiation treatment and trimethylate the same residue. **a** H3K9 acetylation was detected in hippocampal stem cells obtained from brains of WT C57BL/6 mice by immunostaining after 6 Gy IR. **b** H3K9 acetylation was detected in adult murine testis stem cells by immunostaining after 6 Gy IR. **c** H3K9 trimethylation was detected in hippocampal stem cells obtained from brains of WT C57BL/6 mice by immunostaining after 6 Gy IR. **d** H3K9 trimethylation was detected in adult murine testis stem cells by immunostaining after 6 Gy IR. Scale bar = 10 µm. Original picture included for illustrative purposes, referring to ref. ^24^

**Fig. 6:**
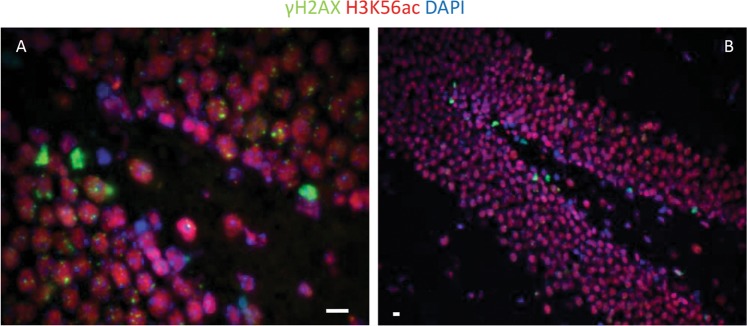
Stem cells display higher levels of H3K56ac (red signals) and pan-nuclear apoptotic H2AX-S139 phosphorylation 6 h after irradiation. H3K56 acetylation and H2AX phosphorylation was detected in hippocampal stem cells obtained from brains of C57BL/6 mice by immunostaining **a** 3 h and **b** 6 h after 6 Gy IR. Scale bar = 10 µm. Original picture included for illustrative purposes, referring to ref. ^23^

Recently, the relationship between histone modifications and stem cell radiation responses has led to new insights: acetylation and methylation on different residues of H3 histone has been shown to play an important role in the radiosensitivity of stem cells. H3K9 modifications have been previously analyzed in ES cells^141–143^. Local downregulation of H3K9ac has been reported in ES cells, accompanying recruitment of the transcription factor OCT4 to DNA lesions^144^. In contrast, we found that ES cells are unable to deacetylate H3K9 at the DNA damage site compared to differentiated cells (Fig 5a, b), thereby limiting trimethylation on the same amino acid residue and consequent DNA damage repair as has been published before^24^ (Fig. 5c, d). In fact, H3K9 trimethylation is fundamental in the regulation of ATM activation after DNA DSB^145,146^. Deacetylation of H3K9 and consequent trimethylation has been proven to increase stem cells radioresistance, decrease IR-induced apoptotic response, and induce DDR activation^24^. Moreover, constitutively elevated histone-3 lysine-56 acetylation (H3K56ac) in stem cells results in a repressive chromatin environment that interferes with DDR activation and promotes radiosensitivity. Recruitment of histone deacetylases and deacetylation of H3K56ac is required at DSB for adequate DDR activation in non-stem cells^147,148^. We have observed higher H3K56 acetylation levels exclusively in neural stem cells of the dentate gyrus displaying elevated H3K56ac^23^ (Fig 6a, b). Knockdown of H3K56 acetyltransferase p300 reduced H3K56ac and significantly decreased radiosensitivity, restored DDR function, and increased stem cell survival^23^.

A constitutively elevated level of H2AX-Y142 phosphorylation has been found in stem cells compared to differentiated counterparts^23^. The relationship between γ-H2AX IRIF, pan-γ-H2AX, and H2AX-pY142 has been previously elucidated^149^, suggesting that the close proximity of persistent H2AX-pY142 sterically hinders access to the S139 site in stem cells, thereby inhibiting DDR signaling and promoting IR-induced apoptosis.

Alterations in methylation levels after 3 Gy of IR have been reported in vivo^150–152^ and a correlation between global DNA hypomethylation and increased radiosensitivity has been observed in somatic cell lines^127,153–155^. On the contrary, exposure to 3 Gy X-irradiation does not lead to changes in DNA methylation in murine ES cells and radiosensitivity is independent of DNA methylation levels. However, de novo methyltransferases DNMT3A and DNMT3B may play a role in modulating sensitivity to X-rays in mESCs as their absence seems to have a modest radioprotective effect^156^. Moreover, DNA methylation status of 50 Long Interspersed Nuclear Element 1 (LINE-1) did not differ significantly in HSCs, hematopoietic progenitor cells, and mononuclear cells in C57BL/6J compared to radiosensitive CBA/J mice immediately after IR. In contrast, a significant decrease in LINE-1 DNA methylation in HSCs was observed in CBA/J 2 months post-treatment, suggesting that epigenetic alterations may potentially serve as driving forces of radiation-induced carcinogenesis^157^.

**Fig. 7:**
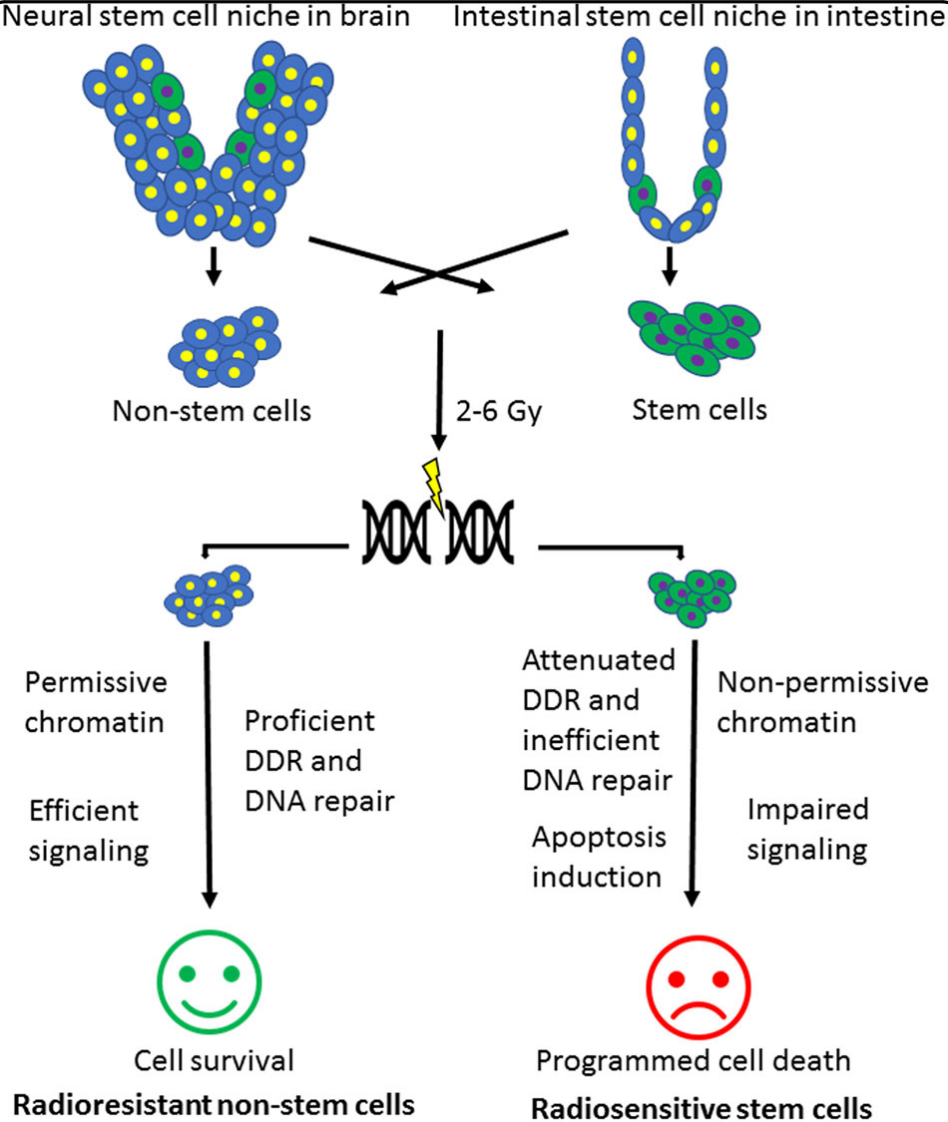
Graphical abstract summarizing the concept of this review. Stem cells reside inside tissue niches together with differentiated non-stem cells. After radiation therapy, non-stem cells respond with chromatin modifications which allows the damage site to become accessible to DDR and repairosome complex to repair the DNA damage. On the contrary, stem cells chromatin configuration remains non-permissive and DNA repair factors are not recruited on damage site. This attenuated DDR, impaired signaling, and induction of apoptosis leads to radiation hypersensitivity of stem cells

5-Hydroxylmethylcytosine (5hmC) is a potential indicator of active DNA demethylation and its distribution patterns provide a global view of gene activation. Murine pluripotent cells have been reported to have constitutively high levels of 5hmC^158,159^ together with elevated γ-H2AX foci^160^ while only a minor subset of γ-H2AX foci colocalized with 5hmC in human embryonic stem cells^161,162^. There is no correlation between 5hmC and radiation response in stem cells. While the role of epigenetics in stem cell pluripotency maintenance and self-renewal as well as differentiation has been largely investigated^163^, further investigations are needed to elucidate its role in stem-cell-specific IR response (Fig. 7).

## Conclusions

Radiation therapy treats many types of cancer effectively, but like other treatments, it can cause complications. Tissue injuries are observed in many cancer patients undergoing IR treatment and the preservation of healthy cells has become an important medical concern, especially in pediatric oncology. Stem cell transplants, although effective in hematopoietic cancer patients, have enormous practical limitations and therefore the protection of in loco radiosensitive stem cells seems to be a more feasible strategy. Our present knowledge on mechanisms of normal stem cell radiosensitivity is still evolving in a piecemeal manner, but clearly the stem cell-specific responses to IR exposure involve molecular genetic, signaling, and epigenetic regulations. Discovering the pluralistically interacting mechanisms and regulatory molecular targets involved in normal stem cell radiosensitivity could lead to innovative therapies that can eradicate cancer while preserving the stem/progenitor cells.

## References

[1] Orthwein A (2014). Mitosis inhibits DNA double-strand break repair to guard against telomere fusions. Science.

[2] Biehs R (2017). DNA double-strand break resection occurs during non-homologous end joining in G1 but is distinct from resection during homologous recombination. Mol. Cell.

[3] Tsouroula K (2016). Temporal and spatial uncoupling of DNA double strand break repair pathways within mammalian heterochromatin. Mol. Cell.

[4] Campisi J, d'Adda di Fagagna F (2007). Cellular senescence: when bad things happen to good cells. Nat. Rev. Mol. Cell Biol..

[5] Edinger A, Thompson C (2004). Death by design: apoptosis, necrosis and autophagy. Curr. Opin. Cell Biol..

[6] Heydarirad G, Rezaeizadeh H, Choopani R, Mosavat SH, Ameri A (2017). Efficacy of a traditional Persian medicine preparation for radiation-induced xerostomia: a randomized, open-label, active-controlled trial. J. Integr. Med..

[7] Ryan JL (2012). Ionizing radiation: the good, the bad, and the ugly. J. Invest. Dermatol..

[8] McQuestion M (2006). Evidence-based skin care management in radiation therapy. Semin. Oncol. Nurs..

[9] Hymes SR, Strom EA, Fife C (2006). Radiation dermatitis: clinical presentation, pathophysiology, and treatment 2006. J. Am. Acad. Dermatol..

[10] Chang DW, te Marvelde L, Chua BH (2014). Prospective study of local control and late radiation toxicity after intraoperative radiation therapy boost for early breast cancer. Int. J. Radiat. Oncol. Biol. Phys..

[11] Hoeller U, Bonacker M, Bajrovic A, Alberti W, Adam G (2004). Radiation-induced plexopathy and fibrosis. Is magnetic resonance imaging the adequate diagnostic tool?. Strahlenther. Onkol..

[12] Dorr W, Hendry JH (2001). Consequential late effects in normal tissues. Radiother. Oncol..

[13] Hellman S, Botnick L (1977). Stem cell depletion: an explanation of the late effects of cytotoxins. Int. J. Radiat. Oncol. Biol. Phys..

[14] Thotala DK, Geng L, Dickey AK, Hallahan DE, Yazlovitskaya EM (2010). A new class of molecular targeted radioprotectors: GSK-3beta inhibitors. Int. J. Radiat. Oncol. Biol. Phys..

[15] Kim HW (2018). Dual effects of human placenta-derived neural cells on neuroprotection and the inhibition of neuroinflammation in a rodent model of Parkinson's disease. Cell Transplant..

[16] Eve DJ (2018). Reduction of microhemorrhages in the spinal cord of symptomatic ALS mice after intravenous human bone marrow stem cell transplantation accompanies repair of the blood-spinal cord barrier. Oncotarget.

[17] Thomsen, G. M. et al. Transplantation of neural progenitor cells expressing glial cell line-derived neurotrophic factor into the motor cortex as a strategy to treat amyotrophic lateral sclerosis. *Stem Cells*10.1002/stem.2825 (2018). 10.1002/stem.2825 29656478

[18] Yin Y (2018). The homing of human umbilical cord-derived mesenchymal stem cells and the subsequent modulation of macrophage polarization in type 2 diabetic mice. Int. Immunopharmacol..

[19] Mucci, A.et al. iPSC-derived macrophages effectively treat pulmonary alveolar proteinosis in Csf2rb-deficient mice. *Stem Cell Rep*. 10.1016/j.stemcr.2018.07.006 (2018). 10.1016/j.stemcr.2018.07.006 PMC6135208 30100408

[20] Zhang, D. Y. et al. Sirtuin3 protects aged human mesenchymal stem cells against oxidative stress and enhances efficacy of cell therapy for ischaemic heart diseases. *J. Cell Mol. Med.*10.1111/jcmm.13821 (2018). 10.1111/jcmm.13821 PMC6201360 30091830

[21] Acharya MM, Rosi S, Jopson T, Limoli CL (2015). Human neural stem cell transplantation provides long-term restoration of neuronal plasticity in the irradiated hippocampus. Cell Transplant..

[22] Baulch JE (2016). Cranial grafting of stem cell-derived microvesicles improves cognition and reduces neuropathology in the irradiated brain. Proc. Natl Acad. Sci. USA.

[23] Jacobs KM (2016). Unique epigenetic influence of H2AX phosphorylation and H3K56 acetylation on normal stem cell radioresponses. Mol. Biol. Cell.

[24] Meyer B (2016). Histone H3 lysine 9 acetylation obstructs ATM activation and promotes ionizing radiation sensitivity in normal stem cells. Stem Cell Rep..

[25] Solokov, M. & Neumman, R. Human embryonic stem cell responses to ionizing radiation exposures: current state of knowledge and future challenges. *Stem Cells Int*. **2012**, 579104 (2012). 10.1155/2012/579104 PMC3431129 22966236

[26] Filion T (2009). Survival responses of human embryonic stem cells to DNA damage. J. Cell. Physiol..

[27] Acharya M (2010). Consequences of ionizing radiation-induced damage in human neural stem cells. Free Radic. Biol. Med.

[28] Katoh O, Tauchi H, Kawaishi K, Kimura A, Satow Y (1995). Expression of the vascular endothelial growth factor (VEGF) receptor gene, KDR, in hematopoietic cells and inhibitory effect of VEGF on apoptotic cell death caused by ionizing radiation. Cancer Res..

[29] Becker D (2009). Response of human hematopoietic stem and progenitor cells to energetic carbon ions. Int. J. Radiat. Biol..

[30] Fabbrizi MR (2018). Transient PP2A inhibition alleviates normal tissue stem cell susceptibility to cell death during radiotherapy. Cell Death Dis..

[31] Lomax ME, Folkes LK, O'Neill P (2013). Biological consequences of radiation-induced DNA damage: relevance to radiotherapy. Clin. Oncol. (R. Coll. Radiol.).

[32] Close DM, Nelson WH, Bernhard WA (2013). DNA damage by the direct effect of ionizing radiation: products produced by two sequential one-electron oxidations. J. Phys. Chem. A.

[33] Katsube T (2017). Effects of chronic restraint-induced stress on radiation-induced chromosomal aberrations in mouse splenocytes. Mutat. Res..

[34] Balajee AS, Bertucci A, Taveras M, Brenner DJ (2014). Multicolour FISH analysis of ionising radiation induced micronucleus formation in human lymphocytes. Mutagenesis.

[35] Fajardo LF (2005). The pathology of ionizing radiation as defined by morphologic patterns. Acta Oncol..

[36] Denham JW, Hauer-Jensen M (2002). The radiotherapeutic injury—a complex 'wound'. Radiother. Oncol..

[37] Kalman NS, Zhao SS, Anscher MS, Urdaneta AI (2017). Current status of targeted radioprotection and radiation injury mitigation and treatment agents: a critical review of the literature. Int. J. Radiat. Oncol. Biol. Phys..

[38] Hall, E. J. & Giaccia, A. J. *Radiobiology for the Radiologist*. 6th edn (Lippincott Williams & Wilkins, 2006).

[39] Withers HR (1967). The dose-survival relationship for irradiation of epithelial cells of mouse skin. Br. J. Radiol..

[40] Withers HR (1971). Regeneration of intestinal mucosa after irradiation. Cancer.

[41] Withers HR, Hunter N, Barkley HT, Reid BO (1974). Radiation survival and regeneration characteristics of spermatogenic stem cells of mouse testis. Radiat. Res..

[42] Withers HR, Mason KA, Thames HD (1986). Late radiation response of kidney assayed by tubule-cell survival. Br. J. Radiol..

[43] McCulloch EA, Till JE (1962). The sensitivity of cells from normal mouse bone marrow to gamma radiation in vitro and in vivo. Radiat. Res..

[44] Gould MN, Biel WF, Clifton KH (1977). Morphological and quantitative studies of gland formation from inocula of monodispersed rat mammary cells. Exp. Cell Res..

[45] Coppes RP, van der Goot A, Lombaert IM (2009). Stem cell therapy to reduce radiation-induced normal tissue damage. Semin. Radiat. Oncol..

[46] Donnelly EH, Smith JM, Farfan EB, Ozcan I (2011). Prenatal radiation exposure: background material for counseling pregnant patients following exposure to radiation. Disaster Med. Public Health Prep..

[47] De Santis M (2007). Radiation effects on development. Birth Defects Res. C Embryo Today.

[48] Brent RL (2015). Protection of the gametes embryo/fetus from prenatal radiation exposure. Health Phys..

[49] Shaw P, Duncan A, Vouyouka A, Ozsvath K (2011). Radiation exposure and pregnancy. J. Vasc. Surg..

[50] Brent RL (1992). Clinical teratology counseling and consultation case report: exposure to diagnostic radiation early in pregnancy. Teratology.

[51] Wakeford R, Little MP (2003). Risk coefficients for childhood cancer after intrauterine irradiation: a review. Int. J. Radiat. Biol..

[52] Momcilovic O (2009). Ionizing radiation induces ataxia telangiectasia mutated-dependent checkpoint signaling and G2 but not G1 cell cycle arrest in pluripotent human embryonic stem cells. Stem Cells.

[53] Suvorova II, Kozhukharova IV, Nikol'skii NN, Pospelov VA (2013). [ATM/ATR signaling pathway activation in human embryonic stem cells after DNA damage]. Tsitologiia.

[54] Sokolov M, Panyutin I, Panyutin I, Neumann R (2011). Dynamics of the transcriptome response of cultured human embryonic stem cells to ionizing radiation exposure. Mutat. Res..

[55] Adams B, Golding S, Rao R, Valerie K (2010). Dynamic dependence on ATR and ATM for double-strand break repair in human embryonic stem cells and neural descendants. PLoS ONE.

[56] Suchorska WM, Augustyniak E, Lukjanow M (2017). Comparison of the early response of human embryonic stem cells and human induced pluripotent stem cells to ionizing radiation. Mol. Med. Rep..

[57] Barazzuol L, Jeggo PA (2016). In vivo sensitivity of the embryonic and adult neural stem cell compartments to low-dose radiation. J. Radiat. Res..

[58] Dumitru R (2012). Human embryonic stem cells have constitutively active Bax at the Golgi and are primed to undergo rapid apoptosis. Mol. Cell.

[59] Mujoo K (2017). Differentiation of human induced pluripotent or embryonic stem cells decreases the DNA damage repair by homologous recombination. Stem Cell Rep..

[60] Wilson K (2010). Effects of ionizing radiation on self-renewal and pluripotency of human embryonic stem cells. Cancer Res..

[61] Yang C (2010). Opposing putative roles for canonical and noncanonical NFkappaB signaling on the survival, proliferation, and differentiation potential of human embryonic stem cells. Stem Cells.

[62] Bai H (2012). Bcl-xL enhances single-cell survival and expansion of human embryonic stem cells without affecting self-renewal. Stem Cell Res..

[63] Conklin J, Baker J, Sage J (2012). The RB family is required for the self-renewal and survival of human embryonic stem cells. Nat. Commun..

[64] Ardehali R (2011). Overexpression of BCL2 enhances survival of human embryonic stem cells during stress and obviates the requirement for serum factors. Proc. Natl Acad. Sci. USA.

[65] Edel M (2010). Rem2 GTPase maintains survival of human embryonic stem cells as well as enhancing reprogramming by regulating p53 and cyclin D1. Genes Dev..

[66] Vinarsky V (2013). Human embryonic and induced pluripotent stem cells express TRAIL receptors and can be sensitized to TRAIL-induced apoptosis. Stem. Cells Dev..

[67] Zhang L (2018). Activated mitochondrial apoptosis in hESCs after dissociation involving the PKA/p-p53/Bax signaling pathway. Exp. Cell Res..

[68] Cmielova J (2012). Gamma radiation induces senescence in human adult mesenchymal stem cells from bone marrow and periodontal ligaments. Int. J. Radiat. Biol..

[69] Ko E, Lee K, Hwang D (2012). Human umbilical cord blood-derived mesenchymal stem cells undergo cellular senescence in response to oxidative stress. Stem. Cells Dev..

[70] Havelek R (2013). Ionizing radiation induces senescence and differentiation of human dental pulp stem cells. Folia Biol. (Krakow).

[71] Sergeeva VA (2017). Low-dose ionizing radiation affects mesenchymal stem cells via extracellular oxidized cell-free DNA: a possible mediator of bystander effect and adaptive response. Oxid. Med. Cell Longev..

[72] Nicolay N (2013). Mesenchymal stem cells retain their defining stem cell characteristics after exposure to ionizing radiation. Int. J. Radiat. Oncol. Biol. Phys..

[73] Nicolay N (2015). Mesenchymal stem cells are resistant to carbon ion radiotherapy. Oncotarget.

[74] Alessio N (2015). Low dose radiation induced senescence of human mesenchymal stromal cells and impaired the autophagy process. Oncotarget.

[75] Li XH, Ha CT, Xiao M (2016). MicroRNA-30 inhibits antiapoptotic factor Mcl-1 in mouse and human hematopoietic cells after radiation exposure. Apoptosis.

[76] Prise KM, Saran A (2011). Concise review: stem cell effects in radiation risk. Stem Cells.

[77] Squillaro T, Galano G, De Rosa R, Peluso G, Galderisi U (2018). Concise review: the effect of low-dose ionizing radiation on stem cell biology: a contribution to radiation risk. Stem Cells.

[78] Fuchs E (2009). The tortoise and the hair: slow-cycling cells in the stem cell race. Cell.

[79] Cheung TH, Rando TA (2013). Molecular regulation of stem cell quiescence. Nat. Rev. Mol. Cell Biol..

[80] Kim JS (2005). Independent and sequential recruitment of NHEJ and HR factors to DNA damage sites in mammalian cells. J. Cell Biol..

[81] Delacote F, Lopez BS (2008). Importance of the cell cycle phase for the choice of the appropriate DSB repair pathway, for genome stability maintenance: the trans-S double-strand break repair model. Cell Cycle.

[82] Mao Z, Bozzella M, Seluanov A, Gorbunova V (2008). Comparison of nonhomologous end joining and homologous recombination in human cells. DNA Repair (Amst.)..

[83] Georgoulis, A., Vorgias, C. E., Chrousos, G. P. & Rogakou, E. P. Genome Instability and gammaH2AX. *Int. J. Mol. Sci*. **18**, 10.3390/ijms18091979 (2017). 10.3390/ijms18091979 PMC5618628 28914798

[84] Chen M (2006). The sensitivity of human mesenchymal stem cells to ionizing radiation. Int. J. Radiat. Oncol. Biol. Phys..

[85] Prendergast A, Cruet-Hennequart S, Shaw G, Barry F, Carty M (2011). Activation of DNA damage response pathways in human mesenchymal stem cells exposed to cisplatin or gamma-irradiation. Cell Cycle.

[86] Oliver L (2013). Differentiation-related response to DNA breaks in human mesenchymal stem cells. Stem Cells.

[87] Wu PK (2017). Early passage mesenchymal stem cells display decreased radiosensitivity and increased DNA repair activity. Stem Cells Transl. Med..

[88] Sokolov M, Neumann R (2013). Lessons learned about human stem cell responses to ionizing radiation exposures: a long road still ahead of us. Int. J. Mol. Sci..

[89] Sugrue T, Lowndes NF, Ceredig R (2014). Hypoxia enhances the radioresistance of mouse mesenchymal stromal cells. Stem Cells.

[90] Islam MS, Stemig ME, Takahashi Y, Hui SK (2015). Radiation response of mesenchymal stem cells derived from bone marrow and human pluripotent stem cells. J. Radiat. Res..

[91] Sugrue T, Lowndes N, Ceredig R (2013). Mesenchymal stromal cells: radio-resistant members of the bone marrow. Immunol. Cell Biol..

[92] Serakinci N (2007). Ectopically hTERT expressing adult human mesenchymal stem cells are less radiosensitive than their telomerase negative counterpart. Exp. Cell Res..

[93] Tsvetkova A (2017). gammaH2AX, 53BP1 and Rad51 protein foci changes in mesenchymal stem cells during prolonged X-ray irradiation. Oncotarget.

[94] Milyavsky M (2010). A distinctive DNA damage response in human hematopoietic stem cells reveals an apoptosis-independent role for p53 in self-renewal. Cell Stem Cell.

[95] Yahata T (2011). Accumulation of oxidative DNA damage restricts the self-renewal capacity of human hematopoietic stem cells. Blood.

[96] Li X (2014). Binding to WGR domain by salidroside activates PARP1 and protects hematopoietic stem cells from oxidative stress. Antioxid. Redox Signal..

[97] Biechonski S (2018). Attenuated DNA damage responses and increased apoptosis characterize human hematopoietic stem cells exposed to irradiation. Sci. Rep..

[98] de Laval B (2013). Thrombopoietin-increased DNA-PK-dependent DNA repair limits hematopoietic stem and progenitor cell mutagenesis in response to DNA damage. Cell Stem Cell.

[99] Aoki H, Hara A, Motohashi T, Kunisada T (2013). Keratinocyte stem cells but not melanocyte stem cells are the primary target for radiation-induced hair graying. J. Invest. Dermatol..

[100] Rachidi W (2007). Sensing radiosensitivity of human epidermal stem cells. Radiother. Oncol..

[101] Shao L, Luo Y, Zhou D (2014). Hematopoietic stem cell injury induced by ionizing radiation. Antioxid. Redox Signal..

[102] Moehrle BM (2015). Stem cell-specific mechanisms ensure genomic fidelity within HSCs and upon aging of HSCs. Cell Rep..

[103] Ishikawa J, Hayashi N, Yamaguchi M, Monzen S, Kashiwakura I (2015). Characteristics of human CD34+ cells exposed to ionizing radiation under cytokine-free conditions. J. Radiat. Res..

[104] Wang Y (2010). Total body irradiation causes residual bone marrow injury by induction of persistent oxidative stress in murine hematopoietic stem cells. Free Radic. Biol. Med..

[105] Wang Y, Kellner J, Liu L, Zhou D (2011). Inhibition of p38 mitogen-activated protein kinase promotes ex vivo hematopoietic stem cell expansion. Stem. Cells Dev..

[106] Chang J (2016). Low doses of oxygen ion irradiation cause acute damage to hematopoietic cells in mice. PLoS ONE.

[107] Miousse IR (2014). Exposure to low-dose (56)Fe-ion radiation induces long-term epigenetic alterations in mouse bone marrow hematopoietic progenitor and stem cells. Radiat. Res..

[108] Katsura M (2016). Effects of chronic low-dose radiation on human neural progenitor cells. Sci. Rep..

[109] Zou Y (2012). Responses of human embryonic stem cells and their differentiated progeny to ionizing radiation. Biochem. Biophys. Res. Commun..

[110] Li, Y. Q., Cheng, Z. & Wong, S. Differential apoptosis radiosensitivity of neural progenitors in adult mouse hippocampus. *Int. J. Mol. Sci*. **17**, 10.3390/ijms17060970 (2016). 10.3390/ijms17060970 PMC4926502 27331809

[111] Ivanov VN, Hei TK (2014). Radiation-induced glioblastoma signaling cascade regulates viability, apoptosis and differentiation of neural stem cells (NSC). Apoptosis.

[112] Ivanov VN, Hei TK (2014). A role for TRAIL/TRAIL-R2 in radiation-induced apoptosis and radiation-induced bystander response of human neural stem cells. Apoptosis.

[113] Isono M (2015). Carbon-ion beams effectively induce growth inhibition and apoptosis in human neural stem cells compared with glioblastoma A172 cells. J. Radiat. Res..

[114] Wang X (2015). Pharmacologically blocking p53-dependent apoptosis protects intestinal stem cells and mice from radiation. Sci. Rep..

[115] Zhu Y, Huang YF, Kek C, Bulavin DV (2013). Apoptosis differently affects lineage tracing of Lgr5 and Bmi1 intestinal stem cell populations. Cell Stem Cell.

[116] Metcalfe C, Kljavin NM, Ybarra R, de Sauvage FJ (2014). Lgr5+ stem cells are indispensable for radiation-induced intestinal regeneration. Cell Stem Cell.

[117] Hua G (2012). Crypt base columnar stem cells in small intestines of mice are radioresistant. Gastroenterology.

[118] Cmielova J (2013). The effect of ATM kinase inhibition on the initial response of human dental pulp and periodontal ligament mesenchymal stem cells to ionizing radiation. Int. J. Radiat. Biol..

[119] Kurpinski K (2009). Differential effects of x-rays and high-energy 56Fe ions on human mesenchymal stem cells. Int. J. Radiat. Oncol. Biol. Phys..

[120] Jin Y (2008). Comprehensive analysis of time- and dose-dependent patterns of gene expression in a human mesenchymal stem cell line exposed to low-dose ionizing radiation. Oncol. Rep..

[121] Liao Y (2016). Paradoxical roles of elongation factor-2 kinase in stem cell survival. J. Biol. Chem..

[122] Gurley KE, Ashley AK, Moser RD, Kemp CJ (2017). Synergy between Prkdc and Trp53 regulates stem cell proliferation and GI-ARS after irradiation. Cell Death Differ..

[123] Wei L (2016). Inhibition of CDK4/6 protects against radiation-induced intestinal injury in mice. J. Clin. Invest..

[124] Tao S (2017). Wnt activity and basal niche position sensitize intestinal stem and progenitor cells to DNA damage. EMBO J..

[125] Hua G (2017). Distinct levels of radioresistance in Lgr5+ colonic epithelial stem cells versus Lgr5+ small intestinal stem cells. Cancer Res..

[126] Chowdhury D (2005). gamma-H2AX dephosphorylation by protein phosphatase 2A facilitates DNA double-strand break repair. Mol. Cell.

[127] Aypar U, Morgan WF, Baulch JE (2011). Radiation-induced genomic instability: are epigenetic mechanisms the missing link?. Int. J. Radiat. Biol..

[128] Kovalchuk O, Baulch JE (2008). Epigenetic changes and nontargeted radiation effects—is there a link?. Environ. Mol. Mutagen..

[129] Li L (2017). Effect of ionizing radiation at low dose on transgenerational carcinogenesis by epigenetic regulation. Lab. Anim. Res..

[130] Song W (2014). Increased p16 DNA methylation in mouse thymic lymphoma induced by irradiation. PLoS ONE.

[131] Yamazaki J (2013). The epigenome of AML stem and progenitor cells. Epigenetics.

[132] Wakita S (2013). Mutations of the epigenetics-modifying gene (DNMT3a, TET2, IDH1/2) at diagnosis may induce FLT3-ITD at relapse in de novo acute myeloid leukemia. Leukemia.

[133] Shih A, Abdel-Wahab O, Patel J, Levine R (2012). The role of mutations in epigenetic regulators in myeloid malignancies. Nat. Rev. Cancer.

[134] Holz-Schietinger C, Matje D, Reich N (2012). Mutations in DNA methyltransferase (DNMT3A) observed in acute myeloid leukemia patients disrupt processive methylation. J. Biol. Chem..

[135] Harikumar A, Meshorer E (2015). Chromatin remodeling and bivalent histone modifications in embryonic stem cells. EMBO Rep..

[136] Gładych M, Andrzejewska A, Oleksiewicz U, Estécio M (2015). Epigenetic mechanisms of induced pluripotency. Contemp. Oncol. (Pozn.).

[137] Shiraki N, Ogaki S, Kume S (2014). Profiling of embryonic stem cell differentiation. Rev. Diabet. Stud..

[138] Kraushaar D, Zhao K (2013). The epigenomics of embryonic stem cell differentiation. Int. J. Biol. Sci..

[139] Liang G, Zhang Y (2013). Embryonic stem cell and induced pluripotent stem cell: an epigenetic perspective. Cell Res..

[140] Ben-David U, Kopper O, Benvenisty N (2012). Expanding the boundaries of embryonic stem cells. Cell Stem Cell.

[141] Etchegaray JP (2015). The histone deacetylase SIRT6 controls embryonic stem cell fate via TET-mediated production of 5-hydroxymethylcytosine. Nat. Cell Biol..

[142] Qiao Y, Wang R, Yang X, Tang K, Jing N (2015). Dual roles of histone H3 lysine 9 acetylation in human embryonic stem cell pluripotency and neural differentiation. J. Biol. Chem..

[143] Hammoud SS (2014). Chromatin and transcription transitions of mammalian adult germline stem cells and spermatogenesis. Cell Stem Cell.

[144] Bartova E (2011). Recruitment of Oct4 protein to UV-damaged chromatin in embryonic stem cells. PLoS ONE.

[145] Ayrapetov MK, Gursoy-Yuzugullu O, Xu C, Xu Y, Price BD (2014). DNA double-strand breaks promote methylation of histone H3 on lysine 9 and transient formation of repressive chromatin. Proc. Natl Acad. Sci. USA.

[146] Sun Y (2009). Histone H3 methylation links DNA damage detection to activation of the tumour suppressor Tip60. Nat. Cell Biol..

[147] Zhu Q (2015). Damaged DNA-binding protein down-regulates epigenetic mark H3K56Ac through histone deacetylase 1 and 2. Mutat. Res..

[148] Miller KM (2010). Human HDAC1 and HDAC2 function in the DNA-damage response to promote DNA nonhomologous end-joining. Nat. Struct. Mol. Biol..

[149] Solier S, Pommier Y (2009). The apoptotic ring: a novel entity with phosphorylated histones H2AX and H2B and activated DNA damage response kinases. Cell Cycle.

[150] Giotopoulos G (2006). DNA methylation during mouse hemopoietic differentiation and radiation-induced leukemia. Exp. Hematol..

[151] Pogribny I, Raiche J, Slovack M, Kovalchuk O (2004). Dose-dependence, sex- and tissue-specificity, and persistence of radiation-induced genomic DNA methylation changes. Biochem. Biophys. Res. Commun..

[152] Wang J (2014). Genome-wide screen of DNA methylation changes induced by low dose X-ray radiation in mice. PLoS ONE.

[153] Dote H (2005). Enhancement of in vitro and in vivo tumor cell radiosensitivity by the DNA methylation inhibitor zebularine. Clin. Cancer Res..

[154] Narayan A, Tuck-Muller C, Weissbecker K, Smeets D, Ehrlich M (2000). Hypersensitivity to radiation-induced non-apoptotic and apoptotic death in cell lines from patients with the ICF chromosome instability syndrome. Mutat. Res..

[155] Aypar U, Morgan WF, Baulch JE (2011). Radiation-induced epigenetic alterations after low and high LET irradiations. Mutat. Res..

[156] Armstrong CA (2012). DNMTs are required for delayed genome instability caused by radiation. Epigenetics.

[157] Miousse, I. R. et al. Inter-strain differences in LINE-1 DNA methylation in the mousehematopoietic system in response to exposure to ionizing radiation. *Int. J. Mol. Sci.* **18**, 10.3390/ijms18071430 (2017). 10.3390/ijms18071430 PMC5535921 28677663

[158] Ruzov A (2011). Lineage-specific distribution of high levels of genomic 5-hydroxymethylcytosine in mammalian development. Cell Res..

[159] Tahiliani M (2009). Conversion of 5-methylcytosine to 5-hydroxymethylcytosine in mammalian DNA by MLL partner TET1. Science.

[160] Banath JP (2009). Explanation for excessive DNA single-strand breaks and endogenous repair foci in pluripotent mouse embryonic stem cells. Exp. Cell Res..

[161] Kafer GR (2016). 5-Hydroxymethylcytosine marks sites of DNA damage and promotes genome stability. Cell Rep..

[162] Takashima Y (2014). Resetting transcription factor control circuitry toward ground-state pluripotency in human. Cell.

[163] Festuccia N, Gonzalez I, Navarro P (2017). The epigenetic paradox of pluripotent ES cells. J. Mol. Biol..

